# Does minimally invasive surgery reduce anxiety?

**DOI:** 10.4274/jtgga.galenos.2018.2018.0073

**Published:** 2019-08-28

**Authors:** Evrim Bostancı Ergen, Yaşam Kemal Akpak, Çetin Kılıççı, Çiğdem Abide Yayla, Selçuk Ayas

**Affiliations:** 1 Clinic of Gynecology Obstetrics and Reproductive Medicine, İstanbul Zeynep Kamil Woman and Child Diseases Training and Research Hospital, İstanbul, Turkey; 2 Clinic of Gynecology Obstetrics and Gynecology, University of Health Sciences, Tepecik Training and Research Hospital, İzmir, Turkey

**Keywords:** Preoperative, postoperative, anxiety, total abdominal hysterectomy, total laparoscopic hysterectomy

## Abstract

**Objective:**

To evaluate whether there were any differences in preoperative and postoperative anxiety in patients who underwent total laparoscopic hysterectomy (TLH) (n=37) and total abdominal hysterectomy (TAH) (n=37).

**Material and Methods:**

All premenopausal patients who underwent TLH or TAH because of benign uterine disorders were enrolled. Anxiety status was assessed 6 hours before and after the operation using standardized validated questionnaires: State-Trait Anxiety Inventory.

**Results:**

In the TAH group, the state anxiety level of the patients significantly increased, whereas there was a significant decrease in the TLH group. For the trait anxiety level, there was a statistically significant increase in the TAH group postoperatively. In the TLH group, trait anxiety levels decreased postoperatively. In the analysis of between-group differences, pre and postoperative the state anxiety level was higher in the TAH group. A statistically significant difference was determined between the groups in respect of the postoperative state anxiety levels (p<0.05), but not in the preoperative state anxiety levels (p>0.05). Statistically significant differences were determined between the groups in respect of education, occupation, and curettage rates (p<0.05).

**Conclusion:**

Women undergoing TLH for benign uterine disease may have lower levels of preoperative and postoperative anxiety than women undergoing TAH.

## Introduction

Hysterectomy is the second most common major surgical procedure applied to women of reproductive age and 90% of the procedures are for benign causes (1). It is known that most patients experience anxiety and fear at different levels before surgery and that anxiety increases during the operation (2,3). However, gynaecologic operations are specific to women, who are more sensitive and emotional so they constitute a specific study group. In this situation, patients fear that their body image will be destroyed, there are concerns related to sexuality, they are anxious about pain, there is the fear of not waking from anaesthesia, and a concern of loss of function (2). Just as much as the effect of anxiety on the patient’s emotional state, anaesthesia complications such as nausea and vomiting have a negative effect on postoperative healing and length of hospital stay (4).

As the measurement and evaluation of anxiety is a difficult subject, it has been halted by many obstructions. Anxiety is a personal issue, so generalization could perpetuate an error and although it can be measured with questions and surveys in patients who are conscious, in those who are unconscious, even if it can be evaluated metabolically, it may not always be possible to reveal objective data (5). The most widely used test in medicine for the measurement of anxiety is the State-Trait Anxiety Inventory (STAI), which was developed by Spielberger et al. (6) and Öner (7).

Although abdominal and vaginal hysterectomies have been performed for many years, laparoscopic hysterectomy was first reported in 1989 (8). There has been increasing interest in more minimally invasive surgical procedures during the past 20 years. In comparison with laparotomy, laparoscopic surgery has some advantages including lower rates of wound infections, shorter hospital stay, and a rapid return to work (9). There are studies comparing psychological well-being, sexuality, and quality of life after laparoscopic and abdominal hysterectomy (10,11,12), and there are studies comparing preoperative and postoperative anxiety in surgeries (13,14). However, to the best of our knowledge, there are no data showing how laparoscopic and abdominal hysterectomy affects pre and postoperative anxiety.

The aim of this study was to evaluate the effect of the type of surgery on the level of anxiety of the patient, through measurements of preoperative and postoperative anxiety levels and an evaluation of the factors affecting these levels.

## Material and Methods

In this prospective comparative study, a total of 74 hysterectomies were performed on patients who met the study inclusion criteria between January 1^st^, 2013, and October 31^st^, 2016. Approval for the study was granted by the Local Ethics Committee.

This study was registered with the www.clinicaltrials.gov protocol registration system (NCT02938845). The patients were classified in two groups; group 1 included patients who underwent total laparoscopic hysterectomy (TLH) (n=37) and group 2 comprised patients who underwent total abdominal hysterectomy (TAH) surgery (n=37). The technical aspects of both types of hysterectomy were discussed with each patient, and the appropriate hysterectomy type was selected through mutual discussion. Anxiety was measured using the STAI questionnaire. The study was designed with 2 assessment points: 6 hours before surgery and 6 hours after surgery. All questionnaires were coded with an identifying number, and the respondents could not view their previous answers. Sample size calculation determined that 33 participants in each group would be sufficient to detect a significant difference on the STAI-TX2, when the mean STAI was set at 37 in the TAH group and at 37 in the TLH group, with an effect size of 0.309 for STAI, difference between standard deviations was 0.19, and difference between means was 0.91.

All hysterectomies were performed for benign reasons. Patients with malignancy, chronic opioid or non-steroidal anti-inflammatory drug use, or chronic pain conditions, a history of two or more caesarean sections, a history of abdominal surgery, autoimmune disease, a history of psychiatric disease, coagulation disorders, the presence of any known systemic or psychiatric disease, those receiving any regular sedative medication at the time of the procedure, those with intraoperatively diagnosed adnexal pathology requiring subsequent unilateral or bilateral oophorectomy, those taking preoperative or postoperative hormone-therapy, and those who were not able to communicate in Turkish were excluded from the study.

Preoperatively, all patients underwent gynecologic examination, medical histories were obtained, and transvaginal ultrasound and routine laboratory tests were performed. All procedures were performed by the same two equally skilled and experienced surgeons (>100 TLH and TAH surgeries) using an identical technique. Informed consent was obtained and all patients were admitted to hospital 1 day preoperatively. General anesthesia was used in all cases and all patients received preoperative antibiotic prophylaxis and anticoagulants during immobilization. All the patients were administered with the same postoperative analgesic procedure (Diclofenac 75 mg/3 mL solution for injection).

The STAI measures both state and trait anxiety. State anxiety (STAI-S) refers to a temporary emotional state related to a specific situation, whereas trait anxiety (STAI-T) represents anxiety as a relatively stable personality characteristic. Each scale has values ranging from 20 to 80, with higher scores representing more severe anxiety. The STAI has no established categories, but a cut-off score of 40 has been used to identify patients with high/very high anxiety. Validity and reliability studies of the Turkish versions of these instruments have been performed (7).

### Statistical analysis

All statistical procedures were performed using SPSS 17.0 for Windows and Microsoft Excel 2010 software. The baseline characteristics of the participants are described with frequency analysis, where scale means are stated as mean ± standard deviation. The chi-square test was used to assess differences between demographic parameters. The Kolmogorov-Smirnov test was used to test the normality of distribution of data in the parameters. Between-group comparisons were made using the independent samples t-test, and the paired-samples t-test was used for within-group differences (pre-after tests). In the evaluation of demographic group- based differences, the independent samples t-test was used for two groups, and one-way ANOVA for more than two groups. The Levene test was used to define the homogeneity of variances. In cases where there was no homogenous variance, robust tests (Welch) were applied. A value of p<0.05 was accepted as statistically significant.

## Results

The baseline characteristics of the TLH and TAH groups are shown in Table 1. Statistically significant differences were determined between the groups in respect of education, occupation, and curettage rates (p<0.05).

**Figure 1:**
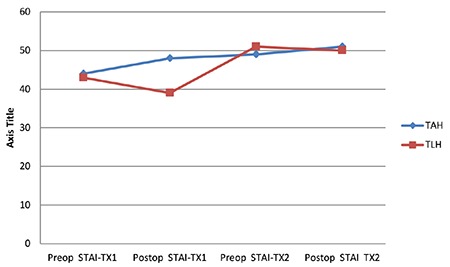
The graph shows the Preop-Postop state and trait anxiety levels between and within TAH and TLH groups TLH: Total laparoscopic hysterectomy; TAH: Total abdominal hysterectomy; STAI-X1: State anxiety scale; STAI-X2: Trait anxiety scale

Between and within-group differences in the preoperative and postoperative state and trait anxiety values are shown in Table 2. The within-group comparisons showed statistically significant differences in the mean TX1 values of both groups (p<0.05). In the TAH group, the state anxiety level (STAI-TX1) significantly increased, and in the TLH group, there was a significant decrease. The trait anxiety level (STAI-TX2) showed a statistically significant increase postoperatively in the TAH group and a decrease in the TLH group. However, this decrease was not statistically significant (p>0.05). In the between-group differences, it was found that the state anxiety level was higher in the TAH group both preoperatively and postoperatively. The postoperative STAI-TX1 differences were found to be statistically significant (p<0.05), but not the preoperative state anxiety differences (p>0.05). In the comparison of STAI-TX2 levels, the preoperative anxiety level was higher in the TLH group, and the postoperative STAI-TX1 level was higher in the TAH group. No statistically significantly difference was determined between the groups in respect of either the preoperative or postoperative STAI-TX2 levels (p>0.05) (Figure 1).

The preoperative state anxiety levels in the TAH group showed statistically significant differences based on occupation and income level of the patient (p<0.05). The postoperative state anxiety levels in the TAH group showed statistically significant differences based on income and the patients’ place of residence (p<0.05). The preoperative trait anxiety level in the TAH group showed statistically significant differences based on income and the patients’ place of residence (p<0.05). The trait anxiety levels in the TLH group showed no statistically significant differences based on the analyzed demographic parameters (p>0.05). The state anxiety levels in the TLH group showed statistically significant differences based on education, marital status, curettage and abortion history of the patients (p<0.05).

## Discussion

In the last decade in particular, TAH and TLH operations have been compared in terms of many factors such as operating time, blood loss during surgery, complication rates, inflammatory response, febrile morbidity, length of stay in hospital, and the requirement for analgesia, but there has been insufficient evaluation in respect of preoperative and postoperative anxiety scores (15). In the current study, although the postoperative patient status (temporary state) and anxiety (general state) were observed to be greater than preoperatively following TAH procedures, a decrease was seen in the postoperative anxiety following TLH procedures compared with the preoperative score. When the postoperative scores were evaluated, a lower anxiety score was determined in the TLH group than in the TAH group. The demographic characteristics were determined to have had a lower effect on TAH procedures than on TLH procedures. It was observed in the TAH group that a higher occupation and income group reduced state anxiety and older age and a rural place of residence reduced trait anxiety. In the TLH group, no factor was observed that affected trait anxiety, but the marital status of the patient, low education levels, and no history of miscarriage or curettage were seen to reduce the level of preoperative state anxiety.

Apart from the several benefits of TLH (e.g., shorter hospital stay, better status on discharge, lower level of postoperative pain) previous studies have not been effective in researching psychological well-being (10,11). In a study that used a visual analogue scale rather than psychometric tests, evaluation was made of scores given from 1-100 daily from preoperative to 35 days postoperatively. From the results obtained, it was determined that there was no superiority of TLH over TAH in respect of patient well-being and mood (16). A meta-analysis conducted in 2014 reported results that were not consistent with the findings of the current study. It was stated that there was no relationship between depression, anxiety, and hysterectomies performed for benign gynaecologic reasons, and it was even reported that hysterectomy reduced symptoms of depression. It was concluded that the type of hysterectomy and surgical technique did not contribute to any psychological effects (17). The main problem of studies in general is the selection of heterogenous patient groups. The current study focussed more on the data of actual anxiety in the short-term, whereas the above-mentioned review evaluated the long-term relief of patients from pain, bleeding, and other symptoms. In the normal female population, the mean anxiety questionnaire evaluation points have been measured as 36.85 (18). The points in the current study were determined to be above this average. However, following TLH, the mean value of the tests evaluating general anxiety was found to be close to this reported average (39.62±5.44).

In a study that researched preoperative risk factors, a history of psychiatric disease, previous diagnosis of cancer, presence of depressive symptoms, history of cigarettte smoking, type of operation, female sex, high level of education, and a history of surgery were evaluated as risk factors for preoperative anxiety (19). Similarly, in the current study, a low education level and no history of curettage were seen to cause a decrease in the preoperative anxiety scores of patients undergoing TLH.

The relationship between age and anxiety has been clearly proven, as it has been demonstrated that younger patients undergoing hysterectomy require more help and experience worse pathologic trauma (20). In the current study, older age was determined as a parameter that reduced trait anxiety in TAH procedures. Other previous studies have observed that married patients felt less anxiety post-hysterectomy due to the support of their husband (21). In the current study, although not at a statistically significant level, this was observed in the scores of the TAH group, whereas the opposite was determined in the TLH group.

A study that was conducted related to previous gynaecologic operations reported that anxiety was increased in patients who had previously felt pain (4), and in the current study, preoperative anxiety was reduced most notably in the TLH group in patients who had not previously undergone curettage. The main reasons for preoperative anxiety were found to be female sex and no history of surgery in studies that researched the reasons for preoperative anxiety in elective surgery (5). However, in contrast to the results of that review, which was conducted on general operations, in the current study of gynaecologic procedures in particular, no history of dilatation and curettage was evaluated as a reason for reduced anxiety.

That the anxiety scores in the preoperative patient group were determined to be higher than the average of the normal population can be attributed to a more unstable psychological structure in gynaecology patients, despite reports stating the opposite and this renders the selection of surgical method more important (2,17). If the type of procedure is evaluated regarding parameters other than psychometric tests, a study in Denmark reported that the hysterectomy type and histopathologic diagnosis had no affect on the prevalence of chronic pelvic pain and this was found to be related more to the personal perception of pain from neurologic nerve damage (22). In a prospective, randomized, multicentre study, laparoscopically assisted vaginal hysterectomy was found to be statistically significantly superior to TAH in respect of early postoperative pain, length of stay in hospital, and patient satisfaction (23).

In a prospective study of 119 patients, a difference was determined between TLH and TAH in respect of psychometric evaluations conducted 5 weeks and 6 months postoperatively. In the same study, in contrast to general evidence, less anxiety and better emotional well-being was observed compared with the preoperative status at the same time points (11). In the current study, the patients were examined in a more acute phase and at close time points and lower anxiety scores were determined in the TLH group than in the TAH group when the postoperative scores were evaluated. Postoperative anxiety scores were also determined to be lower than the preoperative scores in the TLH group.

It can be concluded that laparoscopic surgery should be applied to selected patients because it has positive effects on reducing postoperative anxiety. There is a need for further, prospective studies of larger patient groups to determine which type of hysterectomy causes the least preoperative and postoperative anxiety.

## Figures and Tables

**Table 1:**
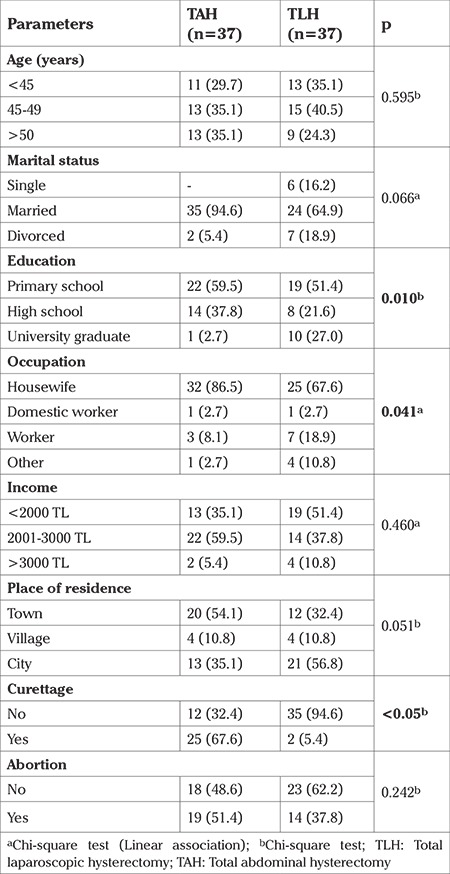
Baseline characteristics of the TAH and TLH groups

**Table 2:**
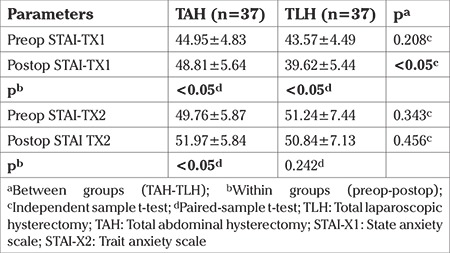
Preop-postop state and trait anxiety levels between and within group differences
